# A Unique Prototypic Device for Radiation Therapy: The p53-Independent Antiproliferative Effect of Neutron Radiation

**DOI:** 10.32607/20758251-2019-11-3-99-102

**Published:** 2019

**Authors:** D. I. Yurkov, S. V. Syromukov, V. V. Tatarskiy, E. S. Ivanova, A. I. Khamidullina, M. A. Yastrebova, V. I. Sysoev, R. V. Dobrov, A. V. Belousov, V. N. Morozov, M. A. Kolyvanova, G. A. Krusanov, V. I. Zverev, A. A. Shtil

**Affiliations:** N.L. Dukhov All-Russia Research Institute of Automatics, Sushchevskaya Str. 22, Moscow, 127055 , Russia; Blokhin National Medical Center of Oncology, Kashirskoye Sh. 24, Moscow, 115478, Russia; Institute of Gene Biology, Russian Academy of Sciences, Vavilova Str. 34/5, Moscow, 119334, Russia; A.I. Burnasyan Federal Medical Biophysical Center, Marshala Novikova Str. 23, Moscow, 123098, Russia; Moscow State University, Department of Physics, Leninskie Gory Str. 1, bldg. 2, Moscow, 119234 , Russia; D.V. Skobeltsyn Institute of Nuclear Physics at Moscow State University, Leninskie Gory Str. 1, bldg. 2, Moscow, 119234, Russia

**Keywords:** fast neutrons, neutron generators, tumor cells, DNA damage, cell death

## Abstract

Radiation therapy with heavy particles including neutrons, an otherwise therapeutically perspective because of its high tissue penetration and efficient tumor damage, is currently limited by the lack of adequate equipment. An NG-24 generator (140 kg, 42 × 110 cm, ~1011 particles/s, > 14 MeV) has been designed and engineered to replace the huge and environmentally harmful neutron reactors, cyclotrons, and accelerators with a compact, portable, safe, and potent source of high-energy neutrons. We demonstrate that the neutron beam produced by NG-24 causes a significant antiproliferative effect on human tumor cell lines regardless of the status of the anti-apoptotic p53 protein. Phosphorylation of histone 2A and increased amounts of p21, cyclin D, and phospho-p53 were detectable in HCT116 colon carcinoma cells (wild-type p53) irradiated with 4 Gy several days post-treatment, accompanied by G2/M phase arrest. These treatments dramatically reduced the ability of single cells to form colonies. In the HCT116p53KO subline (p53 -/-), the G2/M arrest was independent of the aforementioned mechanisms. Hence, the NG-24 generator is a source of a powerful, therapeutically relevant neutron flux that triggers a p53-independent antiproliferative response in tumor cells.

## INTRODUCTION

Photons with an energy range of 30 keV–25 MeV are routinely used in the conventional radiation therapy of tumors. However, heavy particles (in particular, neutrons with energy > 1 MeV; fast neutrons)) are more effective than photons. Due to its high linear energy transfer and relative biological effectiveness (RBE), fast neutron therapy of radioresistant tumors has an advantage over photon beam radiation therapy [1]. Fast neutrons can be used in combination with photon beam therapy. Despite their therapeutic potential, the clinical use of neutrons remains limited, partly because of a lack of adequate equipment. The cyclotrons, nuclear reactors, and accelerators currently in use worldwide [1, 2] are huge stationary devices that are difficult to operate and maintain.

Fast neutron generators can be an alternative. An NG-24 generator designed at the N.L. Dukhov All- Russia Research Institute of Automatics is a compact, portable, and safe device with a therapeutically sufficient particle flux [3]. The characteristics of the NG- 24 generator are shown in Table. Because of its small dimensions, NG-24 can be mounted both on a gantry and on a robotic manipulator. A tritium-saturated target to which a deuterium ion beam accelerated in the electric field is focused serves as a source of irradiation. The nuclear reaction 3H(d,n) generates 14–15 MeV neutrons. Due to a big reaction cross section (5 barn at 107 keV), one can obtain a flux of >1011 neutrons/s [4, 5]. Theoretical calculations and experiments showed that the neutron energy (14.71–14.87 MeV) was practically linearly dependent on the accelerating voltage [6, 7].

**Table 1:** Parameters of the NG-24 neutron source

D-T neutron flux, particles/s	~10^11^
Neutron energy, MeV	> 14
Time resource, h	500
Dimensions, mm	420 × 1100
Weight, kg	140
Electric supply	50/60 Hz, 220 V, 1200 W
Body	Grounded

In this study, we evaluated the ability of a neutron beam produced by NG-24 to induce therapeutically relevant effects in cultured human tumor cells.

## EXPERIMENTAL

The dose absorbed by the cell monolayer was assessed according to the particle flux by computer simulation using the Geant4 software [8] (Physicslist QGSP_BIC_ HP for neutrons with energy < 20 MeV). The calculated coefficient of neutron flux conversion into the dose absorbed by the cell monolayer was 5.7 × 10-13 Gy/neutron. Non-homogeneity of the neutron flux across the monolayer was ± 15%.

The reagents were purchased from PanEco (Russia), except when specified otherwise. The HCT116 colon cancer (wild type p53) and MCF7 human breast cancer (caspase-3 deletion) cell lines were purchased from the American Type Culture Collection. The HCT116p53KO subline with an inactivated p53 protein has been generated in the B. Vogelstein’s laboratory [9]. The cells (50% monolayer) in 25-cm2 cell culture flasks were irradiated with 14 MeV neutrons. For colony formation, 1,000 irradiated cells were plated onto a 100-mm Petri dish in 20 ml of a Dulbecco modified Eagle’s medium supplemented with 10% fetal calf serum (HyClone, USA), 2 mM L-glutamine, 100 U/ml penicillin, and 100 μg/ml streptomycin, and incubated at 37°C, 5% CO_2_ for 14 days. The colonies were fixed with methanol and stained with methyl violet. For flow cytometry and immunoblotting, the irradiated cells were incubated in the aforementioned medium for 1–4 days. The antibodies were purchased from Cell Signaling (USA). The protocols have been published in our earlier papers [10-12].

## RESULTS AND DISCUSSION

Irradiation of HCT116 cells with a single dose of 2 Gy significantly reduced the proliferation rate of the cells; only small individual colonies (≤ 6 cells) were detected after 3 Gy (Figure A). Two days post-irradiation, the cells accumulated in the G2/M phase. This arrest was observed for at least 4 days (Fig. B; note the increased percentage of polyploid cells, a hallmark of altered mitosis). These data indicated that DNA damage was the major mechanism of cell response to the neutron irradiation generated by NG-24. Indeed, after 4 h, the percentage of cells with phosphorylated H2A histone, a marker of DNA double-strand breaks, increased from 10% in untreated cells to > 70% in the cells exposed to 4 Gy. This effect persisted for at least 24 h after irradiation.

**Fig. 1:**
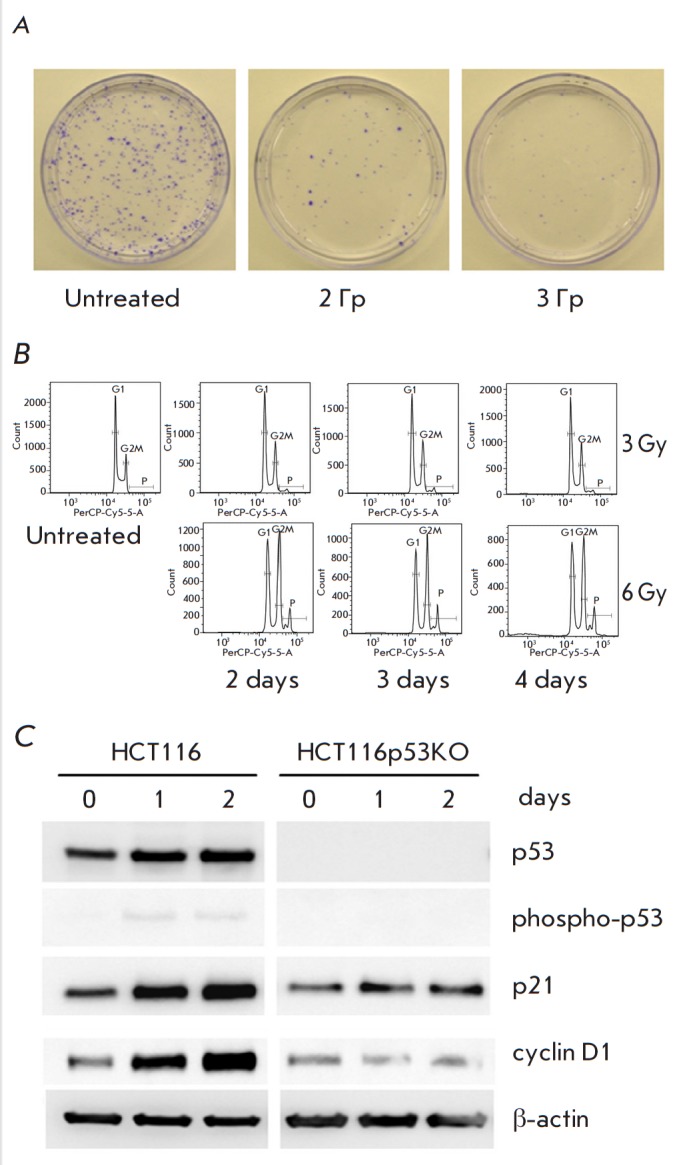
Molecular mechanisms of the response of HCT116 and HCT116p53KO cells to irradiation with fast neutrons. A – colony formation; B – cell cycle distribution (flow cytometry; P – polyploids); C – immunoblotting. The results of at least three replicates are shown

Importantly, the effects of neutron irradiation on HCT116 cells were reproduced for the isogenic subline HCT116p53KO. This subline with inactivated pro-apoptotic p53 is resistant to a number of DNAdamaging anticancer drugs [10]. Therefore, p53 is not needed for the antiproliferative activity of neutrons. The described effects were also observed in a MCF7 breast cancer cell line (caspase-3 deletion). Hence, antiproliferative potency is revealed in cells of different tissue origins; individual nonfunctional mechanisms of cell death, which may limit the therapeutic effects in other situations, do not impede the antitumor efficacy of the neutrons.

The molecular events in cells with the wild type and inactivated p53 were different. The accumulation of p21, the protein known to halt cell cycle progression in response to DNA damage, and activation of cyclin D1 driving the G1-S transition were detected only in HCT116 cells but not in the HCT116p53KO subline (4 Gy; Figure B). Therefore, the increased p53, p21, and cyclin D1 levels contributed to G2/M arrest in HCT116 cells, while other mechanisms are responsible for the same cell cycle arrest in the case of non-functional p53. These mechanisms need to be elucidated, and the final outcome of neutron irradiation (apoptosis, mitotic catastrophe, senescence, etc.) has to be determined.

Hence, the NG-24 neutron generator produces a neutron flux that is sufficient for inducing molecular and phenotypic changes at doses and time intervals relevant to those used in radiation therapy. Therefore, one may expect that the generator can be used in therapeutic applications. Meanwhile, neutron radiation requires special measures for patient safety.

The damage to non-tumor cells caused by neutrons is a crucial issue. Taking into account the dependence between antitumor efficacy and radiation parameters, the lack of information about the biological mechanisms of neutron irradiation, as well as the challenges associated with accurate quantification of the neutronabsorbed dose, there is little sense in comparing the responses of tumor and non-tumor cells. Experimental studies are complicated by the inability of non-transformed cells to form colonies and by the challenges related to long-term culture.

The conformity of neutron radiation is ensured by proper technical solutions. Modern radiotherapy tools allow one to significantly reduce or avoid the damage to surrounding tissues due to the possibilities offered by treatment planning (calculating the radiationabsorbed dose in the tumor and peritumoral tissue). Since the dose produced by neutrons on the surface (skin) is higher than that inside the tumor (the absorbed dose decreases twofold at a depth of 5–6 cm), the therapy employs multiple field irradiations. The dose accumulates in the tumor as the patient’s body is irradiated at different angles. It is possible to reduce the specific surface dose, while the therapeutically effective dose in the tumor is retained. Multileaf collimators are used for an accurate shaping of the tumor. As the radiation source is rotated around the patient’s body, the collimator leaves move to generate a field for each radiation angle. Irradiation may require many collimator leaf positions. Furthermore, the RBE of neutrons increases with a decrease of neutron energy. It is expected that RBE inside the tissue, where the neutrons are slowed down, will be higher compared to that on the skin. However, taking into account the complexity of measuring the RBE value, this question needs further investigation.

This communication demonstrated that the experimental NG-24 neutron generator produces a neutron flux with biological parameters that are acceptable for antitumor radiotherapy. The problem to be solved next is the design of a therapeutic prototype that would combine the high antitumor efficacy of neutron radiation with radiation therapy conformity and meet the requirements for the safety of patients and personnel.

## CONCLUSIONS

This safe and compact NG-24 neutron generator produces a fast neutron flux which allows one to deliver a radiation dose sufficient for inducing a pronounced antiproliferative response in tumor cells. Loss of DNA integrity and delayed cell cycle progression in response to neutron irradiation are detectable regardless of the status of the pro-apoptotic protein p53. These findings suggest that fast neutrons efficiently eliminate tumor cells in which individual molecular mechanisms that control the cell death/survival balance are not functioning.
